# Circulating *Porphyromonas gingivalis* lipopolysaccharide resets cardiac homeostasis in mice through a matrix metalloproteinase-9-dependent mechanism

**DOI:** 10.1002/phy2.79

**Published:** 2013-10-02

**Authors:** Kristine Y DeLeon-Pennell, Lisandra E de Castro Brás, Merry L Lindsey

**Affiliations:** 1 Department of Physiology and Biophysics, San Antonio Cardiovascular Proteomics Center and Jackson Center for Heart Research, University of Mississippi Medical CenterJackson, Mississippi; 2 Research Service, G.V. (Sonny) Montgomery Veterans Affairs Medical CenterJackson, Mississippi

**Keywords:** Cardiac function, inflammation, matrix metalloproteinase-9, periodontal disease, *Porphyromonas gingivalis*, proteomics

## Abstract

*Porphyromonas gingivalis* lipopolysaccharide (*Pg*-LPS) circulates systemically in over 50% of periodontal disease (PD) patients and is associated with increased matrix metalloproteinase (MMP)-9. We hypothesized that low systemic *Pg*-LPS would stimulate an inflammatory response in the left ventricle (LV) through MMP-9, leading to a decrease in cardiac function. Wild-type (WT) and MMP-9 null mice (4–7 months old) were exposed for 1 or 28 days to low dose *Pg*-LPS or saline (*n* ≥ 6/group). MMP-9 significantly increased in WT mice LV at 1 and 28 days of exposure, compared to control (*P* < 0.05 for both). Fractional shortening decreased subtly yet significantly in WT mice by day 28 (31 ± 1%) compared to control (35 ± 1%; *P* < 0.05), and this decrease was attenuated in null (34 ± 1%) mice. Plasma cardiac troponin I levels were elevated in WT mice at day 28. Macrophage-related factors increased over twofold in WT plasma and LV after day 1 (monocyte chemoattractant protein-5, macrophage inflammatory protein (MIP)-1α, MIP-1γ, stem cell factor, *Ccl12*, *Ccl9*, *Il8rb*, *Icam1*, *Itgb2*, and *Spp1*; all *P* < 0.05), indicating a moderate inflammatory response. Levels returned to baseline by day 28, suggesting tolerance to *Pg*-LPS. In contrast, macrophage-related factors remained elevated in day 28 null mice, indicating a sustained defense against *Pg*-LPS stimulation. Consistent with these findings, LV macrophage numbers increased in both groups at day 1 and returned to baseline by day 28 in the WT mice only. Major histocompatibility complex (MCH) II remained elevated in the null group at day 28, confirming *Pg*-tolerance in the WT. Interestingly Il-1α, a regulator of macrophage immunosuppression, increased in the plasma of WT mice only on day 28, suggesting that Il-1α plays a role in tolerance in a MMP-9-dependent manner. In conclusion, circulating *Pg*-LPS induced tolerance in WT mice, resulting in significant LV changes and subtle cardiac dysfunction. MMP-9 played a major role in the regulation of chronic systemic inflammation and associated cardiac dysfunction.

## Introduction

An estimated 75% of the adult population in the United States has periodontal disease (PD), of which 30% has the most severe form. Several oral health measurements, including number of decayed, missing, or filled teeth, mean probing depth, oral hygiene status, and percentage of sites with bleeding on probing, each significantly associate with cardiovascular disease (CVD) incidence (Kaisare et al. 2007; Tonetti et al. 2007). Meta-analysis of more than 200,000 individuals revealed that PD increases CVD risk by 35%, indicating PD has a significant impact on public health (Humphrey et al. 2008). While the oral health and CVD link is quite strong, the mechanistic link between oral health and CVD has not been made.

PD commonly results from a bacterial infection in the gingival tissue surrounding the teeth. As the infection progresses deeper into the tissue, an inflammatory response is activated to combat the infection. *Porphyromonas gingivalis* is an oral pathogen detected in 80% of periodontal patients (Griffen et al. 1998). *Porphyromonas gingivalis* lipopolysaccharide (*Pg*-LPS) has been shown to increase proinflammatory cytokines (Gitlin and Loftin 2009; Gu et al. 2011). Increased expression and activity of matrix metalloproteinases (MMPs) have also been associated with inflammation. MMPs, most notably MMP-9, correlate with chronic PD and CVD (Gu et al. 2011). MMP-9 plays an important role in the inflammatory stimulus by mediating neutrophil and macrophage infiltration (Luttun et al. 2004; Bradley et al. 2012). For these reasons, appropriate management of systemic circulating inflammation may reduce CVD risk factors.

We know that PD patients have systemic levels of *Pg*-LPS that increase circulating inflammatory cytokine levels. In order to test whether *Pg*-induced activation of inflammatory cytokines can amplify risk of CVD, we hypothesized that low systemic *Pg*-LPS would increase the inflammatory response in the left ventricle (LV), leading to a decrease in cardiac function that would be attenuated by MMP-9 deletion.

## Material and Methods

### Mice

C57BL/6J wild-type (WT) and MMP-9 null male and female mice of 4–7 months of age were used in this study (*n* ≥ 6/group). The MMP-9 null mice are on a C57BL/6J background. The null mice were generated by Zena Werb's laboratory and backcrossed by Lynn Matrisian's laboratory (Kaisare et al. 2007; Humphrey et al. 2008). Mice were kept in a light-controlled environment with a 12:12 h light-dark cycle and given free access to standard mice chow and water. Both WT and MMP-9 null colonies were bred in-house and maintained in the same room, and the groups were examined simultaneously. All animal procedures were approved by the Institutional Animal Care and Use Committee at the University of Texas Health Science Center at San Antonio in accordance with the “Guide for the Care and Use of Laboratory Animals.”

### Experimental design

WT and MMP-9 null mice were divided into four groups: no treatment (*n* = 10/genotype), saline treated (*n* = 5/genotype), acute (1 day) *Pg*-LPS (*n* = 6/genotype), and chronic (28 day) *Pg*-LPS (*n* = 20/genotype) treated. No treatment and saline-treated mice were analyzed for all echocardiogram variables, and no significant differences were detected. Based on this analysis, the no treatment and saline-treated groups were combined as a single control group and compared to *Pg*-LPS treated WT or null mice, for a total of six groups.

### *Pg*-LPS infusions

*Porphyromonas gingivalis*-LPS (0.8 μg/g body weight/day; InvivoGen, San Diego, CA) was continuously infused using Alzet osmotic minipumps (model 2004; Durect, Cupertino, CA) implanted subcutaneously as described previously (Koch et al. 1994). The *Pg*-LPS dose is low, consistent with circulating levels in PD patients, and this model is not a sepsis model. Mice were anesthetized with 1–2% isoflurane in an oxygen mix. No mortality was observed, and the saline-treated group served as the negative technical control for this procedure.

### Echocardiography

Transthoracic echocardiography was performed using the Visual Sonics Vevo 770 system (VisualSonics, Toronto, Canada) with a 30-MHz image transducer. Mice were anesthetized with 1–2% isoflurane in oxygen, and electrocardiogram and heart rate were monitored throughout the imaging procedure. All images were acquired at heart rates >400 bpm to maintain physiological relevance. Measurements were taken from the parasternal long-axis B- and M-mode views. For each parameter, three images from consecutive cardiac cycles were measured and averaged (Lindsey et al. 2006).

### Necropsy

For tissue collection, mice were anesthetized with 1–2% isoflurane in an oxygen mix. Five minutes after heparin administration (4 U/g body weight, i.p.), blood was collected from the common carotid artery and immediately centrifuged for collection of plasma. A 1× proteinase inhibitor (Roche, Indianapolis, IN) was added to the plasma, which was stored at −80°C. Plasma samples (100 μL) were sent to Rules Based Medicine (Austin, TX) for multi-analyte proteomic profiling (Chiao et al. 2011). The coronary vasculature was flushed with cardioplegic solution (69 mmol/L NaCl; 12 mmol/L NaHCO_3_; 11 mmol/L glucose; 30 mmol/L 2,3-butanedione monoxime; 10 mmol/L EGTA; 0.001 mmol/L Nifedipine; 50 mmol/L KCl; and 100 U Heparin in 0.9% saline, pH 7.4). Hearts were resected, and the LV and right ventricle (RV) were separated and weighed individually. The LV was sliced into apex, middle, and base sections. The apex and base sections were placed in individual tubes and stored at −80°C for real-time polymerase chain reaction (RT^2^-PCR) or immunoblotting analyses. The middle section was fixed in 10% zinc formalin for histological examination.

### Real-time RT^2^-PCR

RNA extraction was performed using TRIzol® Reagent and Purelink® RNA (Invitrogen, Grand Island, NY) according to the manufacturer's instructions. RNA levels were quantified using the NanoDrop ND-1000 Spectrophotometer (Thermo Scientific, Lafayette, CO). Reverse transcription of equal RNA content (1 μg) was performed using the RT² First Strand Kit (330401; Qiagen, Germantown, MD). Real-Time RT^2^-PCR gene array for Inflammatory Cytokines and Receptors (PAMM-011A; Qiagen) was performed to quantify mRNA levels. All values were normalized to the hypoxanthine guanine phosphoribosyl transferase 1 (Hprt1) housekeeping gene. Only Hprt1 was used for normalization, because this was the only housekeeping gene that does not change following injury. The mRNA levels for all genes measured are shown in Table 3.

### Protein extraction

Total protein was extracted by homogenizing the samples sequentially in phosphate-buffered saline (PBS) with 1× protease inhibitor cocktail, and in protein extraction reagent type 4 (7 mol/L urea, 2 mol/L thiourea, 40 mmol/L Trizma® base, and the detergent 1% C7BzO; Sigma, St. Louis, MO) with 1× protease inhibitor cocktail. Protein concentrations were determined by the Quick Start™ Bradford Protein Assay (Bio-Rad, Hercules, CA).

### Immunoblotting and ELISA

Plasma protein expression of cardiac troponin I (cTnI) was quantified by immunoblotting (ab19615; 1:1000; Abcam, Cambridge, MA). LV protein expression levels were quantified by immunoblotting using antibodies for MMP-9 (ab38898; 1:1000; Abcam) and major histocompatibility complex (MHC) class II (MABF33; 1:1000; Millipore, Billerica, MA). Total protein (10 μg) was separated on 4–12% Criterion™ XT Bis-Tris gels (Bio-Rad), transferred to a nitrocellulose membrane (Bio-Rad), and stained using MemCode™ Reversible Protein Stain Kit (Thermo Scientific) to verify protein concentration and loading accuracy. After blocking with 5% nonfat milk (Bio-Rad), the membrane was incubated with primary antibody, secondary antibody (PI-1000, 1:5000; Vector Laboratories, Burlingame, CA), and detected with ECL Prime Western Blotting Detection Substrate (GE Healthcare Life Sciences, Pittsburgh, PA) or SuperSignal West Femto Chemiluminescent Substrate (Thermo Scientific). Protein levels were quantified by densitometry using the ImageQuant-TL image analysis software (GE Healthcare). The densitometry of the entire lane of the total protein-stained membrane was used for individual lane loading normalization. The relative expression for each immunoblot was calculated as the densitometry of the protein of interest divided by the densitometry of the entire lane of the total protein-stained membrane.

As a secondary confirmation of the cTnI immunoblotting results, an ELISA for cTnI was performed on undiluted plasma collected from each time point using the Life Diagnostics UltraSensitive ELISA Kit for Mouse Plasma (#0.039-2.5). The ELISA was performed according to manufacturer instructions.

### Histology

The middle section of the LV (mid-papillary region) was embedded in paraffin and sectioned at 5 μm. Immunohistochemistry was conducted using the Vectastain ABC Kit (Vector Laboratories). An antibody specific for macrophages (Mac-3, Cedarlane CL8943AP; 1:100) was used to stain macrophages. HistoMark Black (54-75-00; KPL, Gaithersburg, MD) was used to visualize positive staining, with eosin as a counterstain. Negative controls were incubated with no primary antibody. For each LV section, five 60× magnification images were captured. The percentage of the macrophage area was measured using Image-Pro Plus version 6.2 (Media Cybernetics, Inc., Warrendale, PA).

### Statistical analyses

Data are presented as mean ± SEM. Multiple group comparisons were analyzed by one-way analysis of variance (ANOVA), followed by the Student–Newman–Keuls when the Bartlett's variation test passed, or by the nonparametric Kruskal–Wallis test, followed by Dunn post hoc test when the Bartlett's variation test did not pass. *P* < 0.05 was considered statistically significant.

## Results

### MMP-9 increased twofold after *Pg*-LPS exposure in WT

**Figure 1:**
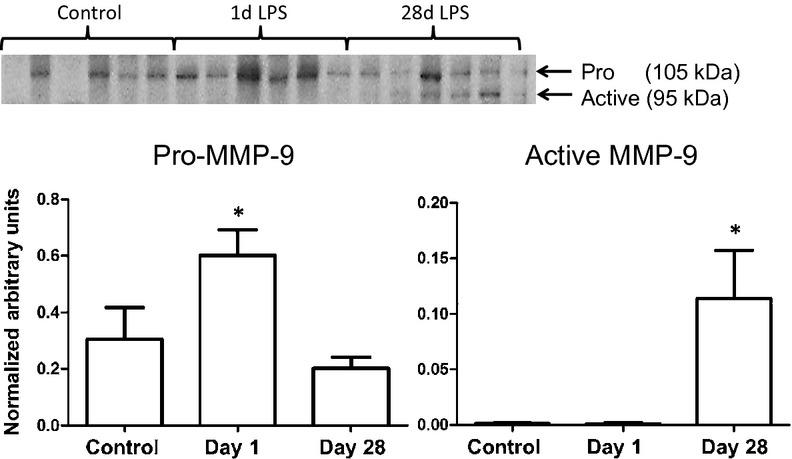
Immunoblotting of MMP-9 in WT and MMP-9 null mice exposed to saline or *Pg*-LPS for 1 or 28 days indicated circulating *Pg*-LPS induces a robust increase of MMP-9 levels in the left ventricle. proMMP-9 levels increased in WT mice with acute exposure to *Pg*-LPS; while active MMP-9 was strongly higher after chronic exposure to *Pg*-LPS. *P < 0.05 versus control, *n* = 6/group.

Matrix metalloproteinase-9 protein levels were determined in the LV of WT animals by immunoblotting. Pro-MMP-9 values doubled after 1 day of *Pg*-LPS exposure (Fig. 1; *P* < 0.05) and returned to baseline levels at day 28. Active MMP-9 protein levels increased by 10-fold compared to control at day 28, demonstrating that chronic exposure to *Pg*-LPS increased MMP-9 activity.

### *Pg*-LPS reduced LV function, and this decrease in function was attenuated in MMP-9 null mice

Cardiac function was measured in WT and MMP-9 null mice using two-dimensional echocardiography (Table 1). MMP-9 null mice showed no overt cardiac dysfunction at baseline, as expected based on previous reports (Ducharme et al. 2000; Lindsey et al. 2006). After 28 days of *Pg*-LPS exposure, fractional shortening decreased slightly yet significantly in WT, but not in null mice. This decrease in fractional shortening was due to an increase in end systolic dimension (ESD), indicating that systolic dysfunction was the driving force for the decline in cardiac function. Wall thinning was not observed in WT or MMP-9 null mice after *Pg*-LPS exposure.

**Table 1:** LV systolic dysfunction occurs in WT mice, but not MMP-9 Null, exposed to *Pg*-LPS

	WT			MMP-9 null		
		
	Control (*n* = 15)	Day 1 (*n* = 6)	Day 28 (*n* = 20)	Control (*n* = 15)	Day 1 (*n* = 6)	Day 28 (*n* = 20)
Heart rate (bpm)	469 ± 9	448 ± 12	445 ± 6	446 ± 6	461 ± 11	454 ± 9
LV wall thickness (mm)	1.23 ± 0.04	1.19 ± 0.03	1.14 ± 0.02	1.25 ± 0.04	1.16 ± 0.04	1.19 ± 0.03
End diasolic dimension (mm)	3.50 ± 0.11	3.22 ± 0.16	3.69 ± 0.07	3.50 ± 0.08	3.21 ± 0.08	3.68 ± 0.08
End systolic dimension (mm)	2.28 ± 0.08	2.05 ± 0.15	2.57 ± 0.07^1^	2.27 ± 0.08	2.00 ± 0.08	2.44 ± 0.07
Fractional shortening%	35 ± 1	37 ± 1	31 ± 1^1^	35 ± 1	38 ± 2	34 ± 1^2^

Values are means ± SEM.

1 *P* < 0.05 versus respective baseline.

2 *P* < 0.05 versus WT 28 day LPS. LV, left ventricle; WT, wild type; MMP-9, matrix metalloproteinase-9; *Pg*-LPS, *Porphyromonas gingivalis* lipopolysaccharide.

### Chronic *Pg*-LPS exposure resulted in myocardial damage in WT, as shown by an increased release of cardiac troponin I in plasma

**Figure 2:**
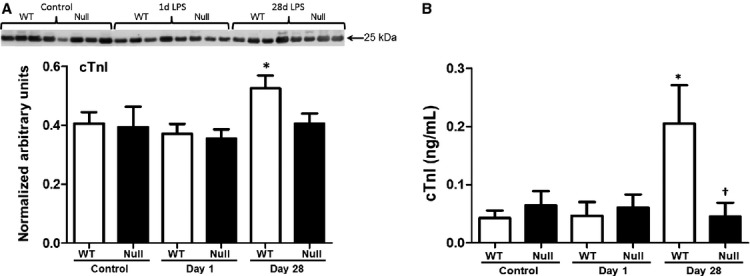
Plasma cardiac troponin I (cTnI) increased in WT at day 28 but not Null as shown by immunoblotting (A) and ELISA (B). *P < 0.05 versus baseline, *n* = 6/group.

In order to further understand the decrease in cardiac function in the WT, plasma levels of cTnI were measured by immunoblotting and ELISA. cTnI is a regulatory protein that controls the calcium-mediated interaction between actin and myosin, and the release of cTnI in the plasma positively correlates with myocardial damage (Adams et al. 1993; Hessel et al. 2008). In the 1 day exposure group, there was no significant change in plasma levels of cTnI in both WT and MMP-9 null mice (Fig. 2). However, by day 28, WT but not null mice showed a slight but significant increase in plasma cTnI indicating chronic exposure of *Pg*-LPS caused myocardial damage consistent with the decrease in the contractility of the myocardium.

### Acute *Pg*-LPS exposure resulted in significant changes at the gene level in LV in both WT and MMP-9 null mice

Out of 84 inflammatory genes analyzed, only six (*Ccl11, Ccl19, Ccl6, Il1r2, Il6st* and *Spp1*) were significantly increased in the LV of both WT and MMP-9 null mice compared to their respective controls (Tables 2 and 3). Of note, these genes were increased only at the 1 day exposure time point. In addition, two genes were decreased at day 1 in both genotypes compared to controls: *Casp1* and *Ccr8*. All eight genes are associated with periodontal health, indicating that the *Pg*-LPS dose given is representative of changes seen in the setting of PD (McGrory et al. 2004; Sharma and Pradeep 2006; Gemmell et al. 2007; Silva et al. 2007).

**Table 2:** Inflammatory Gene Array of WT and MMP-9 null LV. Out of 84 genes evaluated, the 20 shown here were *Pg*-LPS responsive

	WT			MMP-9 null		
		
	Control (*n* = 10)	Day 1 (*n* = 6)	Day 28 (*n* = 10)	Control (*n* = 10)	Day 1 (*n* = 6)	Day 28 (*n* = 10)
*C3*	410.2 ± 69.7	641.1 ± 76.3^a^	395.4 ± 30.5^b^	411.2 ± 44.3	732.4 ± 35.4^d^	689.7 ± 74.1^cd^
*Casp1*	49.4 ± 5.3	31.7 ± 2.2^a^	43.7 ± 3.0	65.4 ± 6.4	42.7 ± 4.2^d^	74.7 ± 6.9^e^
*Ccl11*	9.1 ± 2.1	24.1 ± 4.2^a^	14.9 ± 3.9	15.0 ± 4.2	32.9 ± 4.2^d^	15.9 ± 4.0^e^
*Ccl12*	29.2 ± 6.8	53.3 ± 11.3^a^	19.7 ± 2.1^b^	25.9 ± 4.8	27.3 ± 4.4	39.7 ± 9.9
*Ccl19*	109.8 ± 17.2	189.1 ± 31.2^a^	108.0 ± 10.5^b^	97.6 ± 12.1	210.9 ± 12.1^d^	128.3 ± 18.7^e^
*Ccl25*	8.2 ± 1.4	8.9 ± 1.9	10.2 ± 0.9	8.0 ± 1.0	12.6 ± 0.8^d^	8.3 ± 1.4^e^
*Ccl6*	13.4 ± 2.2	64.8 ± 6.9^a^	15.9 ± 1.8^b^	16.9 ± 2.1	41.4 ± 2.1^b^	19.4 ± 3.0^e^
*Ccl9*	27.6 ± 4.5	45.9 ± 4.6^a^	29.4 ± 2.7^b^	24.1 ± 2.5	37.1 ± 6.1^d^	35.0 ± 2.1^d^
*Ccr3*	16.7 ± 4.9	12.6 ± 3.2	11.5 ± 2.2	18.2 ± 2.1	20.1 ± 1.9	30.7 ± 4.5^cd^
*Ccr8*	1.1 ± 0.3	0.3 ± 0.1^a^	0.5 ± 0.1	0.6 ± 0.2	0.2 ± 0.1^d^	0.5 ± 0.1^e^
*Cxcl1*	5.8 ± 1.4	12.6 ± 3.3^a^	5.8 ± 0.7^b^	7.9 ± 1.2	21.2 ± 2.7^d^	5.6 ± 1.0^e^
*Cxcl13*	1.9 ± 0.5	4.6 ± 1.2	3.2 ± 0.9	0.9 ± 0.2	1.2 ± 0.3	2.5 ± 0.6^d^
*Icam1*	68.9 ± 7.0	110.3 ± 16.7^a^	72.9 ± 5.5^b^	73.3 ± 4.5	74.4 ± 6.9	116.1 ± 6.3^cde^
*Il1r2*	3.8 ± 1.3	7.9 ± 1.2^a^	2.6 ± 0.6^b^	2.5 ± 0.6	5.7 ± 0.4^d^	3.1 ± 0.6^e^
*Il4*	1.3 ± 0.2	0.5 ± 0.1^a^	0.7 ± 0.1^a^	0.6 ± 0.1	0.7 ± 0.2	1.2 ± 0.2^cde^
*Il6ra*	46.2 ± 6.0	51.1 ± 2.5	47.2 ± 2.8	46.2 ± 5.1	65.9 ± 6.4^d^	55.3 ± 3.8^d^
*Il6st*	857.9 ± 74.5	1158.8 ± 49.7^a^	795.9 ± 28.9^b^	587.9 ± 65.3	988.4 ± 84.3^d^	710.6 ± 52.6^e^
*Il8rb*	1.8 ± 0.3	5.8 ± 1.3^a^	3.2 ± 0.7	1.7 ± 0.7	3.9 ± 1.2	3.5 ± 0.9
*Spp1*	0.5 ± 0.3	1.3 ± 0.3^a^	0.4 ± 0.1	0.2 ± 0.2	0.9 ± 0.3^d^	0.5 ± 0.2
*Tnfrsf1b*	36.3 ± 2.5	42.7 ± 3.2	40.0 ± 4.1	30.4 ± 3.5	40.3 ± 6.1	47.1 ± 3.4^d^

^a^Versus WT control, ^b^versus WT 1d, ^c^versus WT 28d, ^d^versus Null control, ^e^versus Null 1d; Values are means ± SEM. *Casp1*, caspase 1; *Ccl*, CC chemokine ligand; *Ccr*, CC chemokine receptor; *Icam1*, intracellular adhesion molecule 1; *Il1r2*, interleukin 1 receptor 2; *Il6st*, interleukin 6 signal transducer; *Spp1*, osteopontin; *Tnfrsf1b*, tumor necrosis factor receptor super family 1b.

### Acute systemic inflammation resulted in increased macrophage numbers in the LV

**Figure 3:**
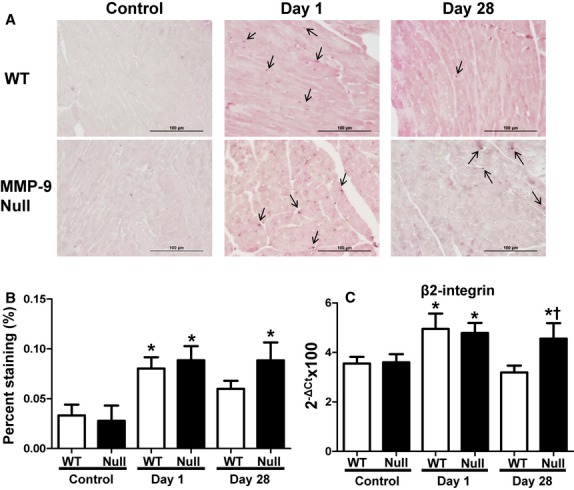
Mac3 staining and *Itgb2* expression of WT and MMP-9 null mice exposed to saline or *Pg*-LPS for 1 or 28 days show macrophages remain elevated in the LV of MMP-9 null but not WT mice, after 28 days exposure to *Pg*-LPS. (A) representative images; (B) quantification of macrophages in each group *n* = 6/group; (C) *Itgb2* mRNA levels in the LV. *P < 0.05 versus baseline, †*P* < 0.05 versus WT day 28, *n* = 6/group.

Macrophage numbers increased at day 1 in both WT and MMP-9 null groups. By day 28, macrophage numbers decreased to baseline levels in the WT but remained elevated in the null mice, giving evidence that MMP-9 deletion provided sustained defense against *Pg*-LPS exposure (Fig. 3). In addition, expression of *Itgb2* in the LV of WT and null mice increased at day 1 but decreased back to baseline levels in the WT and not Null (Fig. 3C).

### Chronic *Pg*-LPS exposure induced macrophage immunosuppression in WT

**Figure 4:**
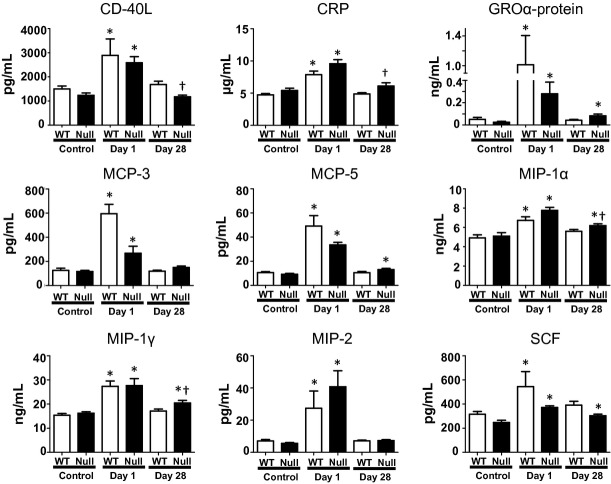
Plasma proteomic profiling of WT and MMP-9 null mice exposed to saline or *Pg*-LPS for 1 or 28 days. Out of 49 analytes evaluated, the 9 shown here were different among groups. *P < 0.05 versus baseline, †*P* < 0.05 versus WT day 28, *n* ≥ 6/group.

To determine how MMP-9 regulated the inflammatory response after *Pg*-LPS exposure, we performed multi-analyte proteomic profiling on plasma and real-time RT^2^-PCR cytokine gene array on the LV (Fig. 4 and Table 2). After 1 day of *Pg*-LPS exposure, several factors related to macrophage recruitment and activation increased at least twofold in WT mice compared to controls (all *P* < 0.05), including plasma analytes: C-reactive protein (CRP), CD40 ligand, growth-regulated α-protein (GROα-protein), macrophage inflammatory protein (MIP)-1α, -1γ, -2, monocyte chemotactic protein (MCP)-3, -5, stem cell factor (SCF), and LV genes: *C3, Ccl12/MCP-5, Ccl9-MIP-1γ, Ccl19/MIP-3β, Cxcl1/GROα, Il8rb, Icam1, Itgb1, and Itgb2* (De Nichilo and Burns 1993; Koch et al. 1994; Mantovani et al. 2004; Okada et al. 2009; Cassetta et al. 2011; Nelson et al. 2011). However, after 28 days of *Pg*-LPS exposure these factors returned to baseline levels in the WT, suggesting immune response suppression or endotoxin tolerance (Roth et al. 1994; West and Koons 2008).

**Table 3:** Inflammatory mRNA levels evaluated in WT and MMP-9 null after exposure to saline or *Pg*-LPS for 1 or 28 days

	WT			MMP-9 null		
		
	Control (*n* = 10)	Day 1 (*n* = 6)	Day 28 (*n* = 10)	Control (*n* = 10)	Day 1 (*n* = 6)	Day 28 (*n* = 10)
*Abcf1*	780.0 ± 43.0	825.1 ± 45.2	756.6 ± 27.6	624.4 ± 66.5	699.2 ± 62.4	669.4 ± 50.9
*Bcl6*	125.7 ± 11.2	118.7 ± 14.1	139.5 ± 13.9	130.5 ± 15.2	81.5 ± 9.4^d^	116.8 ± 5.7^e^
*C3*	410.2 ± 69.7	641.1 ± 76.3^a^	395.4 ± 30.5^b^	411.2 ± 44.3	732.4 ± 35.4^d^	689.7 ± 74.1^cd^
*Casp1*	49.4 ± 5.3	31.7 ± 2.2^d^	43.7 ± 3.0	65.4 ± 6.4	42.7 ± 4.2^d^	74.7 ± 6.9^e^
*Ccl11*	9.1 ± 2.1	24.1 ± 4.2^a^	14.9 ± 3.9	15.0 ± 4.2	32.9 ± 4.2^d^	15.9 ± 4.0^e^
*Ccl12*	29.2 ± 6.8	53.3 ± 11.3^a^	19.7 ± 2.1^b^	25.9 ± 4.8	27.3 ± 4.4	39.7 ± 9.9
*Ccl17*	1.3 ± 0.3	1.3 ± 0.5	1.6 ± 0.2	1.1 ± 0.2	0.5 ± 0.1	1.1 ± 0.3
*Ccl19*	109.8 ± 17.2	189.1 ± 31.2^a^	108.0 ± 10.5^b^	97.6 ± 12.1	210.9 ± 12.1^d^	128.3 ± 18.7^e^
*Ccl2*	52.8 ± 9.2	56.8 ± 12.7	53.7 ± 7.6	68.5 ± 7.0	69.2 ± 12.5	81.7 ± 8.2
*Ccl22*	1.2 ± 0.3	0.3 ± 0.1^a^	0.9 ± 0.2^b^	0.8 ± 0.2	0.4 ± 0.1	0.8 ± 0.2
*Ccl24*	2.9 ± 0.7	1.9 ± 0.5	3.3 ± 0.5	2.1 ± 0.4	1.2 ± 0.4	3.2 ± 0.5
*Ccl25*	8.2 ± 1.4	8.9 ± 1.9	10.2 ± 0.9	8.0 ± 1.0	12.6 ± 0.8^d^	8.3 ± 1.4^e^
*Ccl3*	1.4 ± 0.3	1.5 ± 0.3	0.8 ± 0.2	1.6 ± 0.2	0.9 ± 0.3	2.3 ± 0.4^c^
*Ccl4*	2.7 ± 0.6	2.4 ± 0.4	2.6 ± 0.4	5.2 ± 0.8^a^	4.8 ± 0.7^b^	5.3 ± 0.7^c^
*Ccl5*	16.6 ± 2.7	7.7 ± 0.6^a^	12.9 ± 1.4	28.4 ± 2.7^a^	19.6 ± 2.8^b^	30.0 ± 3.2^c^
*Ccl6*	13.4 ± 2.2	64.8 ± 6.9^a^	15.9 ± 1.8^b^	16.9 ± 2.1	41.4 ± 2.1^d^	19.4 ± 3.0^e^
*Ccl7*	34.4 ± 8.7	73.5 ± 21.5	30.5 ± 4.6	47.2 ± 8.8	85.4 ± 16.0	53.9 ± 10.9
*Ccl8*	11.1 ± 2.7	18.3 ± 5.0	11.8 ± 1.7	16.9 ± 4.9	29.1 ± 4.2	18.1 ± 2.7
*Ccl9*	27.6 ± 4.5	45.9 ± 4.6^a^	29.4 ± 2.7^b^	24.1 ± 2.5	37.1 ± 6.1^d^	35.0 ± 2.1^d^
*Ccr1*	12.2 ± 2.9	15.6 ± 2.7	9.6 ± 1.4	10.4 ± 2.0	10.7 ± 2.5	11.2 ± 1.2
*Ccr10*	32.1 ± 5.2	15.8 ± 2.1	21.9 ± 4.5	22.1 ± 2.8	20.8 ± 1.8	32.7 ± 2.7^de^
*Ccr2*	9.1 ± 2.6	11.0 ± 2.7	9.6 ± 1.2	16.7 ± 2.5	18.6 ± 1.7	27.9 ± 4.4^c^
*Ccr3*	16.7 ± 4.9	12.6 ± 3.2	11.5 ± 2.2	18.2 ± 2.1	20.1 ± 1.9	30.7 ± 4.5^cd^
*Ccr5*	23.5 ± 5.4	25.3 ± 4.8	15.7 ± 2.9	21.9 ± 2.9	38.8 ± 9.3	29.1 ± 4.2
*Ccr7*	4.8 ± 0.6	1.4 ± 0.3^a^	2.5 ± 0.5^a^	4.6 ± 0.9	2.3 ± 0.5	4.3 ± 0.7
*Ccr8*	1.1 ± 0.3	0.3 ± 0.1^a^	0.5 ± 0.1	0.6 ± 0.2	0.2 ± 0.1^d^	0.5 ± 0.1^e^
*Ccr9*	10.1 ± 1.9	6.0 ± 0.9	8.3 ± 1.7	6.8 ± 0.8	7.3 ± 0.6	11.5 ± 1.7
*Cx3 cl1*	114.0 ± 8.3	71.9 ± 4.7^a^	89.9 ± 4.1	95.2 ± 7.3	91.8 ± 8.2	121.8 ± 12.7
*Cxcl1*	5.8 ± 1.4	12.6 ± 3.3	5.8 ± 0.7	7.9 ± 1.2	21.2 ± 2.7^d^	5.6 ± 1.0^e^
*Cxcl10*	5.8 ± 1.4	6.2 ± 2.0	6.2 ± 1.1	16.5 ± 2.5	8.1 ± 1.4	17.5 ± 4.3
*Cxcl11*	1.2 ± 0.3	0.8 ± 0.1	1.6 ± 0.4	1.9 ± 0.4	1.1 ± 0.3	1.9 ± 0.3
*Cxcl12*	1188.2 ± 106.2	1055.2 ± 105.2	1062.3 ± 102.6	1277.0 ± 67.8	1308.4 ± 41.4	1414.9 ± 39.9^c^
*Cxcl13*	1.9 ± 0.5	4.6 ± 1.2	3.2 ± 0.9	0.9 ± 0.2	1.2 ± 0.3	2.5 ± 0.6^d^
*Cxcl5*	2.6 ± 0.5	2.9 ± 0.5	1.7 ± 0.5	2.3 ± 0.4	4.0 ± 1.0	3.2 ± 0.6
*Cxcl9*	35.3 ± 5.4	13.6 ± 2.3^a^	24.5 ± 4.2	38.8 ± 11.1	21.2 ± 4.4	39.4 ± 8.3
*Cxcr3*	2.2 ± 0.5	0.8 ± 0.3^a^	1.4 ± 0.2	1.7 ± 0.4	2.7 ± 0.7^b^	3.4 ± 0.6
*Cxcr5*	2.4 ± 0.4	1.0 ± 0.1	1.6 ± 0.4	1.7 ± 0.4	1.2 ± 0.4	2.0 ± 0.5
*Icam1*	68.9 ± 7.0	110.3 ± 16.7^a^	72.9 ± 5.5^b^	73.3 ± 4.5	74.4 ± 6.9	116.1 ± 6.3^cde^
*Il10*	1.6 ± 0.3	3.0 ± 0.3^a^	1.5 ± 0.4^b^	2.1 ± 0.3	1.7 ± 0.6	2.6 ± 0.6
*Il10ra*	19.7 ± 4.6	11.9 ± 2.0	13.4 ± 1.6	17.0 ± 2.9	16.0 ± 3.0	21.2 ± 1.6
*Il10rb*	1273.0 ± 108.3	1079.6 ± 47.7	1152.9 ± 63.5	944.1 ± 109.5	1162.0 ± 159.5	1014.8 ± 60.1
*Il11*	1.6 ± 0.2	0.7 ± 0.1^a^	1.2 ± 0.2	1.2 ± 0.4	1.0 ± 0.2	1.1 ± 0.3
*Il13ra1*	231.2 ± 15.5	241.9 ± 15.8	206.3 ± 19.1	225.8 ± 21.6	177.9 ± 29.5	202.4 ± 13.4
*Il15*	310.0 ± 27.7	329.8 ± 20.1	265.4 ± 23.5	278.1 ± 31.6	309.9 ± 27.9	296.2 ± 21.4
*Il16*	25.2 ± 3.8	13.5 ± 1.7	22.7 ± 3.7	21.4 ± 3.0	15.2 ± 1.9	27.4 ± 3.0
*Il18*	3.6 ± 1.0	5.0 ± 1.3	5.6 ± 0.6	7.9 ± 1.3	7.8 ± 1.0	7.9 ± 1.0
*Il1a*	1.9 ± 0.3	1.3 ± 0.4	1.3 ± 0.3	1.2 ± 0.3	0.6 ± 0.2	1.0 ± 0.2
*Il1b*	9.9 ± 2.1	7.4 ± 2.4	6.6 ± 1.0	8.5 ± 1.4	11.5 ± 1.7	9.7 ± 2.1
*Il1r1*	97.0 ± 7.3	88.9 ± 4.8	87.7 ± 6.0	79.6 ± 4.0	106.5 ± 11.4^d^	106.5 ± 6.0^d^
*Il1r2*	3.8 ± 1.3	7.9 ± 1.2^a^	2.6 ± 0.6^b^	2.5 ± 0.6	5.7 ± 0.4^d^	3.1 ± 0.6^e^
*Il2rb*	3.6 ± 0.8	1.9 ± 0.4	1.7 ± 0.4	3.3 ± 0.5	2.1 ± 0.4	4.0 ± 0.4^c^
*Il2rg*	85.2 ± 7.2	62.6 ± 3.1^a^	62.4 ± 5.1^a^	82.4 ± 6.6	70.1 ± 3.4	103.6 ± 9.7^c^
*Il4*	1.3 ± 0.2	0.5 ± 0.1^a^	0.7 ± 0.1^a^	0.6 ± 0.1	0.7 ± 0.2	1.2 ± 0.2^cde^
*Il6ra*	46.2 ± 6.0	51.1 ± 2.5	47.2 ± 2.8	46.2 ± 5.1	65.9 ± 6.4^d^	55.3 ± 3.8^e^
*Il6st*	857.9 ± 74.5	1158.8 ± 49.7^a^	795.9 ± 28.9^b^	587.9 ± 65.3	988.4 ± 84.3^d^	710.6 ± 52.6^e^
*Il8rb*	1.8 ± 0.3	5.8 ± 1.3^a^	3.2 ± 0.7	1.7 ± 0.7	3.9 ± 1.2	3.5 ± 0.9
*Itgam*	44.6 ± 8.6	64.9 ± 5.2	33.4 ± 4.2	40.7 ± 4.8	73.7 ± 10.6^d^	57.9 ± 4.9^c^
*Mif*	1590.8 ± 171.0	1430.5 ± 131.8	1561.5 ± 99.6	909.2 ± 162.0	1141.3 ± 118.7	1151.8 ± 153.2
*Pf4*	110.5 ± 14.4	145.3 ± 13.0	87.0 ± 7.2	92.1 ± 15.4	114.2 ± 13.3	113.2 ± 10.5
*Scye1*	871.3 ± 86.5	932.5 ± 60.1	881.4 ± 42.0	624.9 ± 65.6	836.7 ± 47.6	726.6 ± 60.1
*Spp1*	0.5 ± 0.3	1.3 ± 0.3^a^	0.4 ± 0.1	0.2 ± 0.2	0.9 ± 0.3^d^	0.5 ± 0.2
*Tgfb1*	211.9 ± 13.4	178.2 ± 13.2	200.8 ± 15.9	232.5 ± 15.1	185.2 ± 15.1	260.1 ± 17.4^c^
*Tnf*	3.9 ± 1.0	1.4 ± 0.4	2.7 ± 0.5	5.5 ± 0.8	2.6 ± 0.4	6.3 ± 1.2
*Tnfrsf1a*	122.6 ± 15.7	156.6 ± 6.8	95.5 ± 4.5	85.0 ± 14.8	135.7 ± 12.1	112.8 ± 17.4
*Tnfrsf1b*	36.3 ± 2.5	42.7 ± 3.2	40.0 ± 4.1	30.4 ± 3.5	40.3 ± 6.1	47.1 ± 3.4^d^
*Tollip*	296.4 ± 32.8	331.1 ± 8.1	258.1 ± 12.8	218.0 ± 14.0	297.8 ± 29.4^d^	235.4 ± 10.1
*Xcr1*	1.9 ± 0.6	0.6 ± 0.1	1.2 ± 0.3	2.2 ± 0.6	1.2 ± 0.3	2.7 ± 0.6

^a^Versus WT control, ^b^versus WT 1d, ^c^versus WT 28d, ^d^versus Null control, and ^e^versus Null 1d; Values are means ± SEM. Not detectable: *Ccl1*, *Ccl20*, *Ccr4*, *Ccr6*, *Cd40lg*, *CRP*, *Cxcl15*, *Ifng*, *Il13*, *Il17b*, *Il1f6*, *Il1f8*, *Il20*, *Il3*, *Il5ra*, *Lta*, and *Ltb*.

Similar to WT groups, macrophage activation or recruitment plasma analytes (CRP, CD40 ligand, GROα-protein, MIP-1α, -1γ, -2, MCP-3, -5, SCF) and LV genes (*C3, Ccl9/MIP-1γ, Ccl11/eotaxin-1, Ccl19/MIP-3β, Ccl25, Cxcl1/GROα, Il6ra, Icam1, and Itgb2*, *P* < 0.05 for all compared to control) increased in null mice after 1-day exposure. Of these, CRP, GROα-protein, MCP-5, MIP-1α, MIP-1γ, SCF, *C3, Ccl9, Il6ra, and Itgb2* remained elevated with chronic exposure to *Pg-*LPS*,* and *Ccr3, Cxcl13, Il4,* and *Tnfrsf1b* had significantly higher expression levels at day 28 compared to baseline control. That *Ccl9*/MIP-1γ and *Cxcl1*/GROα had a similar pattern at both the systemic and local levels in both WT and MMP-9 null groups give evidence of sustained inflammatory response in the Null LV after chronic exposure to *Pg*-LPS.

**Figure 5:**
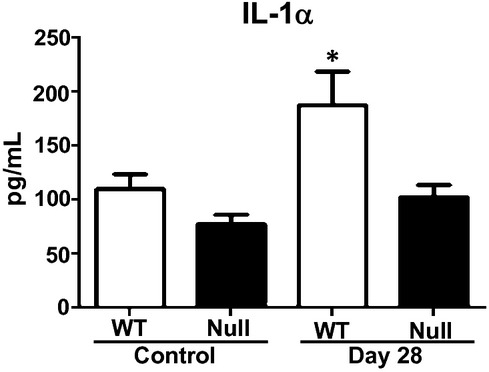
Plasma interleukin-1α (Il-1α) of WT and MMP-9 null mice exposed to saline or *Pg*-LPS for 1 or 28 days show increased levels in WT at day 28 but not Null. *P < 0.05 versus respective baseline, *n* ≥ 6/group.

Interestingly, Il-1α increased in WT but not in null mice as shown in Figure 5. Previous studies have indicated Il-1α to have a role in immunosuppression of macrophages (Tomioka et al. 1996). The increase in plasma Il-1α in WT could explain the reduced macrophage recruitment found in the LV at day 28. Overexpression of these plasma analytes and genes in the null group suggests that MMP-9 deletion has a cardioprotective role against chronic *Pg*-LPS exposure, by sustaining macrophage infiltration and the inflammatory defense system.

### WT mice showed desensitization to the LPS stimulus by day 28

**Figure 6:**
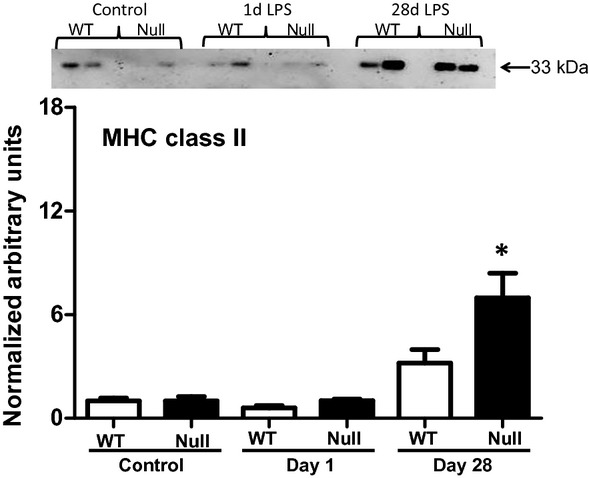
MHC class II immunoblotting of WT and MMP-9 null mice exposed to saline or Pg-LPS for 1 or 28 days reveals MHC class II increased in MMP-9 null but not WT after 28 days *Pg*-LPS exposure. *P < 0.05 versus baseline, *n* = 6/group.

Major histocompatibility complex class II is involved in presentation of extracellular pathogens. Under normal inflammatory conditions, MHC class II expression increase in order to stimulate the immune response. However, during endotoxin tolerance antigen presentation is decreased compared to nontolerant macrophages (Wolk et al. 2000, 2003). We quantified MHC class II protein levels to evaluate if WT mice developed tolerance to *Pg*-LPS exposure, as suggested by the plasma proteomic profiling and LV gene array results. MHC class II protein values were increased in the null (*P* < 0.05) but not in the WT mice at day 28 (Fig. 6). Because MMP-9 null animals showed lower protein levels at baseline, we normalized protein values to their respective baseline values. Lower levels of MHC class II are associated with impaired antigen presentation capacity causing a reduction in the reparative immune response (McGrory et al. 2004; Nelson et al. 2011).

**Figure 7:**
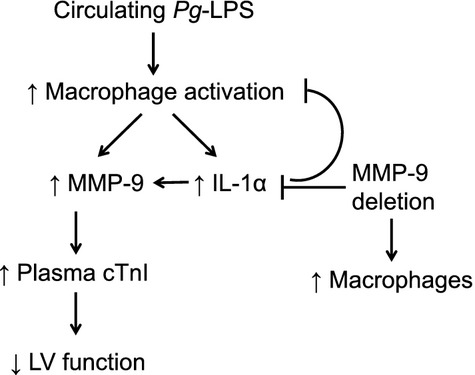
Schematic of MMP-9-dependent *Pg*-LPS effects in cardiac function.

## Discussion

The goal of this study was to investigate the mechanisms that underlie the connection between PD and CVD incidence. Our data show that circulating *Pg*-LPS (1) increased MMP-9 protein levels in the WT LV resulting in myocardial damage and a decrease in LV function, (2) stimulated a strong systemic response after 1 day exposure in the WT and MMP-9 null animals, (3) increased macrophage numbers in the LV of both genotypes, and (4) induced desensitization in the WT group after chronic exposure that was MMP-9-dependent. These data suggest that PD plays a key role in CVD by inducing a strong inflammatory response that is regulated by MMP-9 (Fig. 7).

MMP-9 levels have been correlated with an increase in plasma levels of cTnI (Manginas et al. 2005; Uzuelli et al. 2008), and increased levels of plasma cTnI are associated with myocardial damage (Adams et al. 1993). After 28 days of *Pg*-LPS exposure, our data show that MMP-9 and plasma cTnI increased in WT only. This increase was accompanied by a decrease in fractional shortening, suggesting that *Pg*-LPS exposure induces LV dysfunction through MMP-9 effects on systolic function. This finding is in conflict with findings published in Ashigaki et al., however in this study the exposure to *P. gingivalis* is only 14 days compared to our study which exposed the mice for 28 days to *Pg*-LPS (Ashigaki et al. 2013). This suggests that longer exposure time may be needed to induce cardiac dysfunction through increased MMP-9.

Circulating *Pg*-LPS has been shown to increase the inflammatory response and is believed to be the source of CVD risk in PD patients (Scannapieco et al. 2003; Pussinen et al. 2007). This study showed that the *Pg*-LPS exposure induced a strong inflammatory response in both WT and MMP-9 null. This was evidenced by high levels of inflammatory markers in plasma, and increased expression of proinflammatory cytokines in the LV. Interestingly, plasma and gene levels of inflammatory markers associated with macrophage recruitment and activation returned to baseline values with chronic exposure to *Pg*-LPS in the WT group, but remained elevated in the Null. Although this increase is modest in the Null group, it is sufficient to help the immune system combat the infection without causing myocardial damage.

Consistent with plasma and gene array results acute exposure increased macrophage numbers in both WT and MMP-9 null mice. Continuous exposure to *Pg*-LPS caused lower numbers of macrophages in the WT LV at day 28. This was not observed in the null mice, where values remained elevated from day 1 to day 28 compared to control. The increase in plasma Il-1α observed in the WT mice at day 28 most likely played a role in the decrease on macrophage recruitment/activation after chronic exposure, as Il-1α can induce immunosuppression by causing a decrease in antigen presentation on macrophages after chronic microbial exposure (Tomioka et al. 1996).

Based on these results, chronic exposure to *Pg-*LPS induced endotoxin tolerance or desensitization in the WT group. This was confirmed by an increase in MHC class II protein levels in the LV of MMP-9 null but not WT mice. MMP-9 deletion prevented *Pg*-LPS tolerance by attenuating the increase in plasma Il-1α and increasing MHC class II in the LV after 28 days of exposure, which resulted in a prolonged inflammatory response but better cardiac function.

In conclusion, PD induces increased risk of CVD by altering the inflammatory response in a MMP-9-dependent manner. MMP-9 plays a major role in the regulation of chronic systemic inflammation and associated cardiac dysfunction. Future studies evaluating the effect of preexisting PD on cardiac injury responses are warranted. Most notably phenotyping of the infiltrated cells would allow for better mechanistic insights on how PD induces cardiac injury. It would be interesting to look at leukocytes obtained from infected mice and determine if antigen-presentation and T-cell stimulatory capability were increased by deletion of MMP-9.
